# MiR‐125b but not miR‐125a is upregulated and exhibits a trend to correlate with enhanced disease severity, inflammation, and increased mortality in sepsis patients

**DOI:** 10.1002/jcla.23094

**Published:** 2019-11-07

**Authors:** Xiaoping Zhu

**Affiliations:** ^1^ Departments of Anesthesia The First Affiliated Hospital of Nanchang University Nanchang China

**Keywords:** disease risk, disease severity, miR‐125a, miR‐125b, sepsis

## Abstract

**Objective:**

This study aimed to investigate the correlation of miR‐125a/b expressions with disease risk, progression, and prognosis of sepsis.

**Methods:**

MiR‐125a/b expressions and inflammatory cytokines were detected by RT‐qPCR and ELISA assays in plasma samples from 120 sepsis patients. Besides, blood biochemical indexes, disease severity scores, and in‐hospital mortality of sepsis patients were recorded. Meanwhile, miR‐125a/b expressions in plasma from 120 health controls (HCs) were also detected by RT‐qPCR.

**Results:**

MiR‐125b was elevated in sepsis patients compared with HCs, and ROC curve revealed that miR‐125b could well distinguish sepsis patients from HCs with AUC 0.658. MiR‐125b positively correlated with APACHE II score, SOFA score, Scr, CRP, PCT, TNF‐α, and IL‐6 levels. Most interestingly, miR‐125b was greatly decreased in survivors compared with non‐survivors, and multivariate analysis revealed that miR‐125b independently predicted higher mortality risk in sepsis patients. Besides, miR‐125a showed no significant correlation with sepsis risk, disease severity, or prognosis.

**Conclusion:**

MiR‐125b but not miR‐125a is upregulated and exhibits a trend to correlate with enhanced disease severity, inflammation, and increased mortality in sepsis patients.

## 1 INTRODUCTION

Sepsis is a critical life‐threatening organ dysfunction caused by a dysregulated host response to infection which is characterized by severe inflammation, immune activities, and coagulation.^1^ It is recently disclosed by a global statistics report that approximately above 30 million patients suffer from sepsis per year, and the incidences of hospital‐treated sepsis and severe sepsis are 437 per 100,000 person‐year and 270 per 100 000 person‐year, respectively.^2^ As in China, it is reported that the sepsis incidence is around 37% and sepsis mortality is around 33% in ICU settings, and the latter is approximately onefold higher than the mortality in developed countries.^3^, ^4^, ^5^, ^6^ Considering the abovementioned high incidence, as well as mortality of sepsis, and the delay of diagnosis is a deteriorative issue for worse outcome in sepsis, it is crucial to observe novel biomarkers for early diagnosis, disease progression, or even prognosis.

MiR‐125a and miR‐125b are two common reported critical genes in human physiological and pathological process especially for regulating inflammation, immune activities, and coagulation.^7^, ^8^, ^9^ For instance, miR‐125a and miR‐125b induce inflammation and impair antiviral response via repressing A20 and mitochondrial antiviral signaling (MAVS) in chronic obstructive pulmonary disease (COPD)^8^; miR‐125a and miR‐125b promote coagulation via regulating coagulation factor Ⅸ mini‐gene through inhibiting nonsense‐mediated mRNA decay in mammalian cells.^9^ Combining these above data and that the sepsis is featured by severe inflammation, dysregulated immune activities, and coagulation, we hypothesized that miR‐125a and miR‐125b might possess the potential to be biomarker for sepsis.

Thus in this present study, we aimed to investigate the correlation of miR‐125a/b expressions with sepsis risk, and further explore their association with disease progression and prognosis in sepsis patients.

## 2 MATERIALS AND METHODS

### 2.1 Participants

Between January 2016 and May 2018, 120 sepsis patients and 120 healthy controls (HCs) from The First Affiliated Hospital of Nanchang University were consecutively enrolled in this study. The inclusion criteria for sepsis patients were as follows: (a) diagnosed as sepsis according to the criteria of 2001 SCCM/ESICM/ACCP/ATS/SIS International Sepsis Definitions Conference^10^; (b) age more than 18 years; (c) admitted to intensive care units (ICUs) within 24 hours after onset of symptoms. The exclusion criteria for sepsis were: (a) undergoing immunosuppressive therapy or cancer‐related chemotherapy; (b) had human immunodeficiency virus (HIV) infection; (c) complicated with hematological malignancies or tumors; (d) pregnant or lactating women. For the HCs, the inclusion criteria were: (a) age‐ and gender‐matched to the recruited sepsis patients; (b) without history of sepsis or other severe infections. The exclusion criteria for HCs were as same as the sepsis patients. The present study was approved by the Institutional Review Board of The First Affiliated Hospital of Nanchang University, and all participants or their guardians provided the written informed consents.

### 2.2 Data collection

When the written informed consents were obtained from patients or their guardians, clinical characteristics of all enrolled sepsis were recorded, such as age, gender, body mass index (BMI) smoke, chronic comorbidities including chronic obstructive pulmonary disease (COPD), cardiomyopathy, chronic kidney failure and cirrhosis, serum creatinine (Scr), albumin, white blood cell (WBC), C‐reactive protein (CRP), and procalcitonin (PCT). Besides, acute physiology and chronic health evaluation (APACHE) II score as well as sequential organ failure assessment (SOFA) score assessed within 24 hours after ICU admission were also collected, which were used to evaluate disease severity (higher scores were associated with higher severity). All enrolled sepsis patients were treated in accordance with the guidelines and followed up until mortality in the hospital or discharge, and the number of survivors and non‐survivors were recorded.

### 2.3 Blood samples collection

For the enrolled sepsis patients, blood samples were collected within 24 hours after ICU admission; as for the HCs, blood samples were collected after they signed informed consents. All samples were centrifuged to separate plasma within 1 hour after collection at 4°C 2000 *g* for 10 minutes, then the plasma was scored at −80°C for the further detections of miR‐125a/b and inflammatory cytokines.

### 2.4 Determination of miR‐125a/b

Total RNA was extracted from plasma samples using Trizol Reagent (Invitrogen), then cDNA was reversely transcribed using PrimeScript RT reagent kit (Takara). Real‐time quantitative polymerase chain reaction (RT‐qPCR) was then performed using SYBR Premix Ex TaqTM II (Takara). The relative expressions of miR‐125a/b were calculated using 2^−△△Ct^ method with U6 as internal reference. Primers used in the present study were as follows: miR‐125a, forward: ACACTCCAGCTGGGTCCCTGAGACCCTTTAAC, reverse: TGTCGTGGAGTCGGCAATTC; miR‐125b, forward: ACACTCCAGCTGGGTCCCTGAGACCCTAACTT, reverse: TGTCGTGGAGTCGGCAATTC; U6, forward: CTCGCTTCGGCAGCACATATACTA, reverse: ACGAATTTGCGTGTCATCCTTGC.

### 2.5 Determination of inflammatory cytokines

For the sepsis patients, the levels of inflammatory cytokines including tumor necrosis factor (TNF‐α), interleukin‐1β (lL‐1β), IL‐6, and IL‐17 in plasma were determined using human enzyme‐linked immunosorbent assay (ELISA) kits (Abcam), and all samples processing and operations were carried out in accordance with the kits instructions strictly.

### 2.6 Statistical analysis

SPSS 21.0 software (SPSS Inc) and GraphPad Prism 7.00 software (GraphPad Software) were used for statistical analysis and figures making. Normal distributed continuous variable was presented as mean value ± standard deviation, skewed distributed continuous variable was presented as median (25th‐75th quantiles), and categorized variable was presented as count (percentage). Comparison of skewed distributed continuous variable was determined by Wilcoxon rank sum test; correlation analysis was determined by Wilcoxon rank sum test or Spearman rank correlation test; the diagnostic utility of miR‐125a/b for sepsis and the ability of miR‐125a/b to discriminate between survivor and non‐survivor were assessed by use of receiver operating characteristic (ROC) curve and area under the ROC curve (AUC). Factors predicting mortality were determined by the univariate and multivariate logistic regression model analyses.

## 3 RESULTS

### 3.1 Characteristics

The mean age of sepsis patients was 59.1 ± 12.1 years, and there were 84 (70.0%) males and 36 (30.0%) females. Nineteen (15.8%), 42 (35.0%), 11 (9.2%), and 21 (17.5%) cases were complicated with COPD, cardiomyopathy, chronic kidney failure, and cirrhosis, respectively. The mean APACHE II score and SOFA score were 15.4 ± 6.4 and 7.2 ± 3.5, respectively. The other detailed characteristics were presented in Table 1.

**Table 1:** Characteristics of sepsis patients

Characteristics	Sepsis patients (N = 120)
Age (years)	59.1 ± 12.1
Gender (n/%)	
Male	84 (70.0)
Female	36 (30.0)
BMI (kg/m^2^)	22.6 ± 5.1
Smoke (n/%)	36 (30.0)
Chronic comorbidities (n/%)	
COPD	19 (15.8)
Cardiomyopathy	42 (35.0)
Chronic kidney failure	11 (9.2)
Cirrhosis	21 (17.5)
Scr (mg/dL)	1.7 (1.2‐2.3)
Albumin (g/L)	25.1 (21.2‐34.4)
WBC (10^9^/L)	16.6 (3.0‐28.7)
CRP (mg/L)	100.6 (57.1‐140.9)
PCT (ng/mL)	14.8 (8.6‐28.2)
APACHE II score	15.4 ± 6.4
SOFA score	7.2 ± 3.5
TNF‐α (pg/mL)	192.5 (124.9‐270.7)
IL‐1β (pg/mL)	12.2 (5.6‐24.6)
IL‐6 (pg/mL)	66.5 (41.7‐118.6）
IL‐17 (pg/mL)	142.6 (71.5‐220.6)

Note

Data were presented as mean value ± standard deviation, count (percentage), or median (25th‐75th quantiles).

Abbreviations: APACHE, acute physiology and chronic health evaluation; BMI, body mass index; COPD, chronic obstructive pulmonary disease; CRP, C‐reactive protein; PCT, procalcitonin; Scr, serum creatinine; SOFA, sequential organ failure assessment; TNF, tumor necrosis factor; IL: interleukin; WBC, white blood cell.

### 3.2 Expressions of miR‐125a/b in sepsis patients and HCs

**Figure 1:**
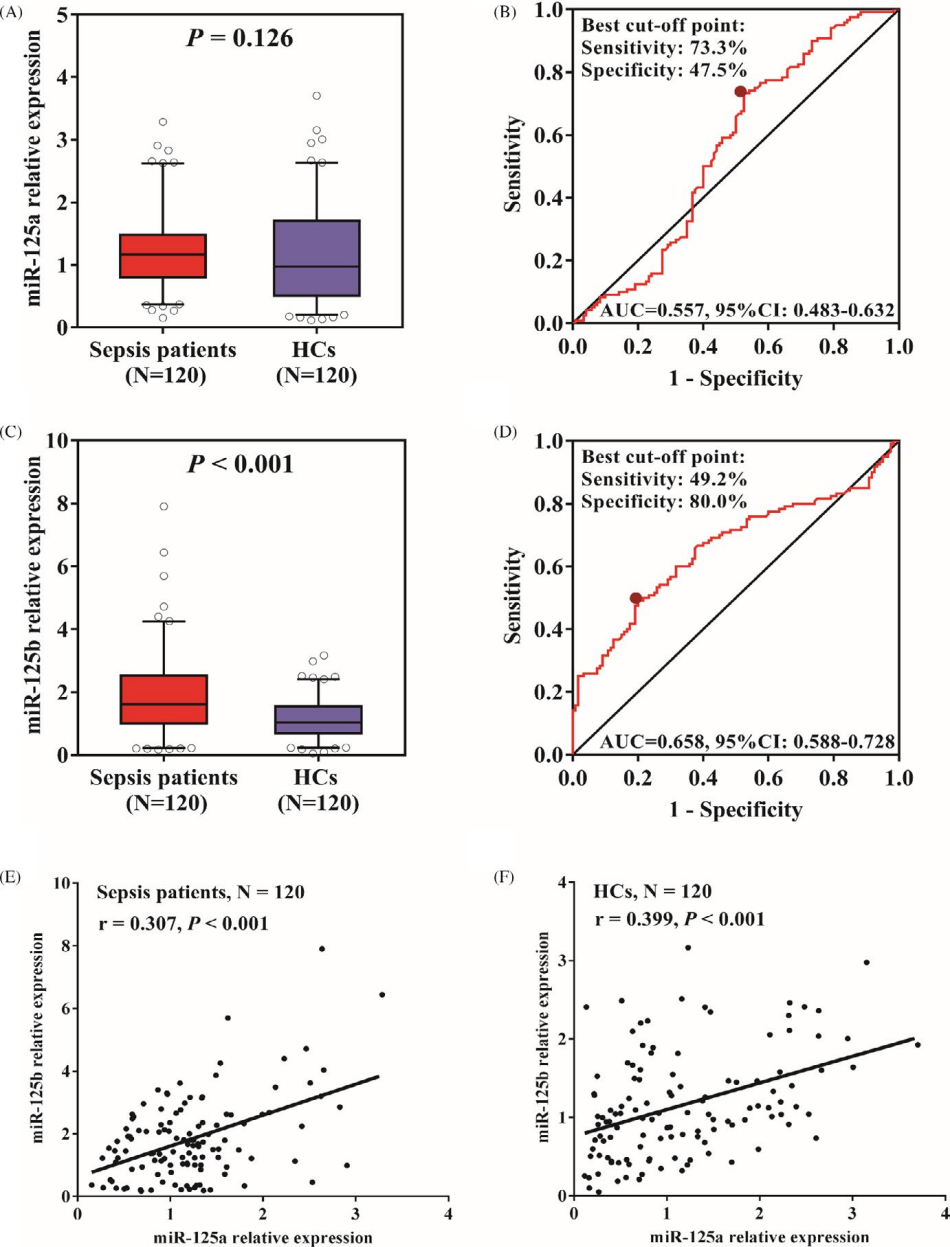
Correlation of miR‐125a/b expressions with sepsis risk. A, miR‐125a expression in sepsis patients and controls. B, The value miR‐125a expression in predicting sepsis risk. C, miR‐125b expression in sepsis patients and controls. D, The value miR‐125b expression in predicting sepsis risk. E, Correlation between miR‐125a and miR‐125b in sepsis patients. F, Correlation between miR‐125a and miR‐125b in HCs. HCs, health controls

MiR‐125a expression showed no difference between sepsis patients and HCs (*P* = .126) (Figure 1A), and ROC curve exhibited miR‐125a could not differentiate sepsis patients from HCs with AUC 0.557 95%CI 0.483‐0.632 (Figure 1B). Interestingly, miR‐125b expression was greatly elevated in sepsis patients compared with HCs (*P* < .001) (Figure 1C), and ROC curve revealed that miR‐125b could well distinguish sepsis patients from HCs with AUC 0.658 95%CI 0.588‐0.728, and the sensitivity and specificity were 49.2% and 80.0% at the best cutoff point which was defined as the point achieved maximum sensitivity plus specificity (Figure 1D). In addition, a positive correlation between miR‐125a and miR‐125b was observed both in sepsis patients (*P* < .001) (Figure 1E) and HCs (*P* < .001) (Figure 1F).

### 3.3 Correlation of miR‐125a/b expressions with APACHE II and SOFA scores in sepsis patients

**Figure 2:**
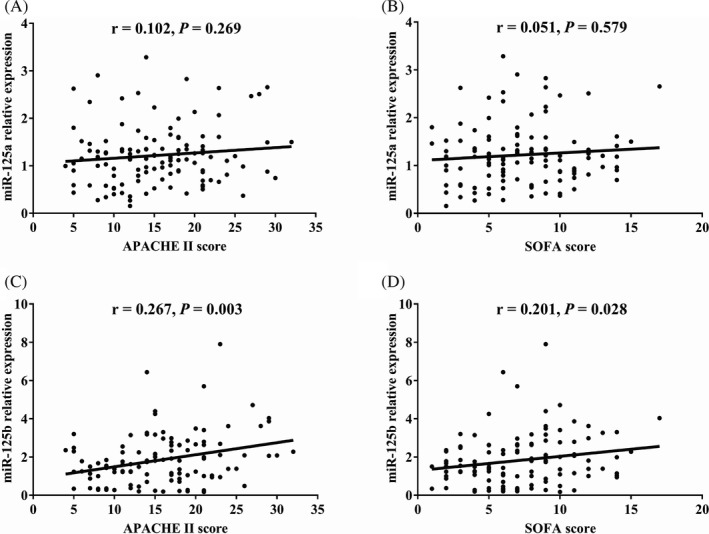
miR‐125b but not miR‐125a positively correlated with disease severity. A, Correlation of miR‐125a expression with APACHE II score. B, Correlation of miR‐125a expression with SOFA score. C, Correlation of miR‐125b expression with APACHE II score. D, Correlation of miR‐125b expression with SOFA score. APACHE II, acute physiology and chronic health evaluation; SOFA, sequential organ failure assessment

Correlation of miR‐125a/b expressions with general disease severity scores including APACHE II and SOFA was analyzed, which showed that miR‐125a was not correlated with APACHE II score (*P* = .269) (Figure 2A) or SOFA score (*P* = .579) (Figure 2B), while miR‐125b was positively associated with APACHE II score (*P* = .003) (Figure 2C) and SOFA score (*P* = .028) (Figure 2D).

### 3.4 Correlation of miR‐125a/b expressions with other characteristics in sepsis patients

Correlation of miR‐125a/b expressions with other characteristics other than APACHE II and SOFA scores was further analyzed, which illuminated that miR‐125a was positively correlated with cardiomyopathy complication (*P* = .015), and miR‐125b was positively correlated with Scr (*P* = .002), CRP (*P* < .001), PCT (*P* = .010), TNF‐α (*P* = .008), and IL‐6 (*P* = .028) levels (Tables 2 and 3).

**Table 2:** Correlation of miR‐125a/b relative expression with categorical variables of sepsis patients

Items	miR‐125a relative expression	*P* value	miR‐125b relative expression	*P* value
Gender				
Male	1.2 (0.8‐1.4)	.968	1.5 (1.0‐2.2)	.731
Female	1.0 (0.8‐1.6)		1.6 (1.0‐2.6)	
Smoke				
Yes	1.2 (0.7‐1.7)	.360	1.9 (1.0‐3.2)	.097
No	1.1 (0.8‐1.4)		1.5 (0.9‐2.3)	
COPD				
Yes	1.2 (0.9‐1.6)	.733	1.8 (1.0‐2.6)	.727
No	1.2 (0.8‐1.4)		1.6 (1.0‐2.4)	
Cardiomyopathy				
Yes	1.2 (1.0‐1.6)	.015	2.1 (1.0‐3.0)	.094
No	1.0 (0.6‐1.4)		1.5 (0.9‐2.1)	
Chronic kidney failure				
Yes	0.9 (0.7‐1.6)	.366	0.8 (0.2‐3.4)	.395
No	1.2 (0.8‐1.5)		1.6 (2.0‐2.4)	
Cirrhosis				
Yes	1.2 (0.8‐1.6)	.785	1.2 (1.0‐1.8)	.195
No	1.1 (0.8‐1.5)		1.8 (0.9‐2.6)	

Note

Data were presented as median (25th‐75th quantiles). Comparison was determined by Wilcoxon rank sum test. *P* value <.05 was considered significant.

Abbreviation: COPD, chronic obstructive pulmonary disease.

**Table 3:** Correlation of miR‐125a/b relative expression with continuous variables of sepsis patients

Items	miR‐125a relative expression		miR‐125b relative expression	
	*P* value	*r*	*P* value	*r*
Age	.181	.123	.773	.027
BMI	.870	.015	.104	−.149
Scr	.226	.111	.002	.276
Albumin	.982	−.002	.809	.022
WBC	.209	.116	.686	.037
CRP	.197	.119	<.001	.387
PCT	.237	.109	.010	.234
TNF‐α	.218	.113	.008	.243
IL‐1β	.723	.033	.159	.129
IL‐6	.475	.066	.028	.200
IL‐17	.964	.004	.051	.179

Note

Correlation was determined by Spearman rank correlation test. *P* value <.05 was considered significant.

Abbreviations: BMI, body mass index; CRP, C‐reactive protein; IL, interleukin; PCT, procalcitonin; Scr, serum creatinine; TNF, tumor necrosis factor; WBC, white blood cell.

### 3.5 Correlation of miR‐125a/b expressions with mortality in sepsis patients

**Figure 3:**
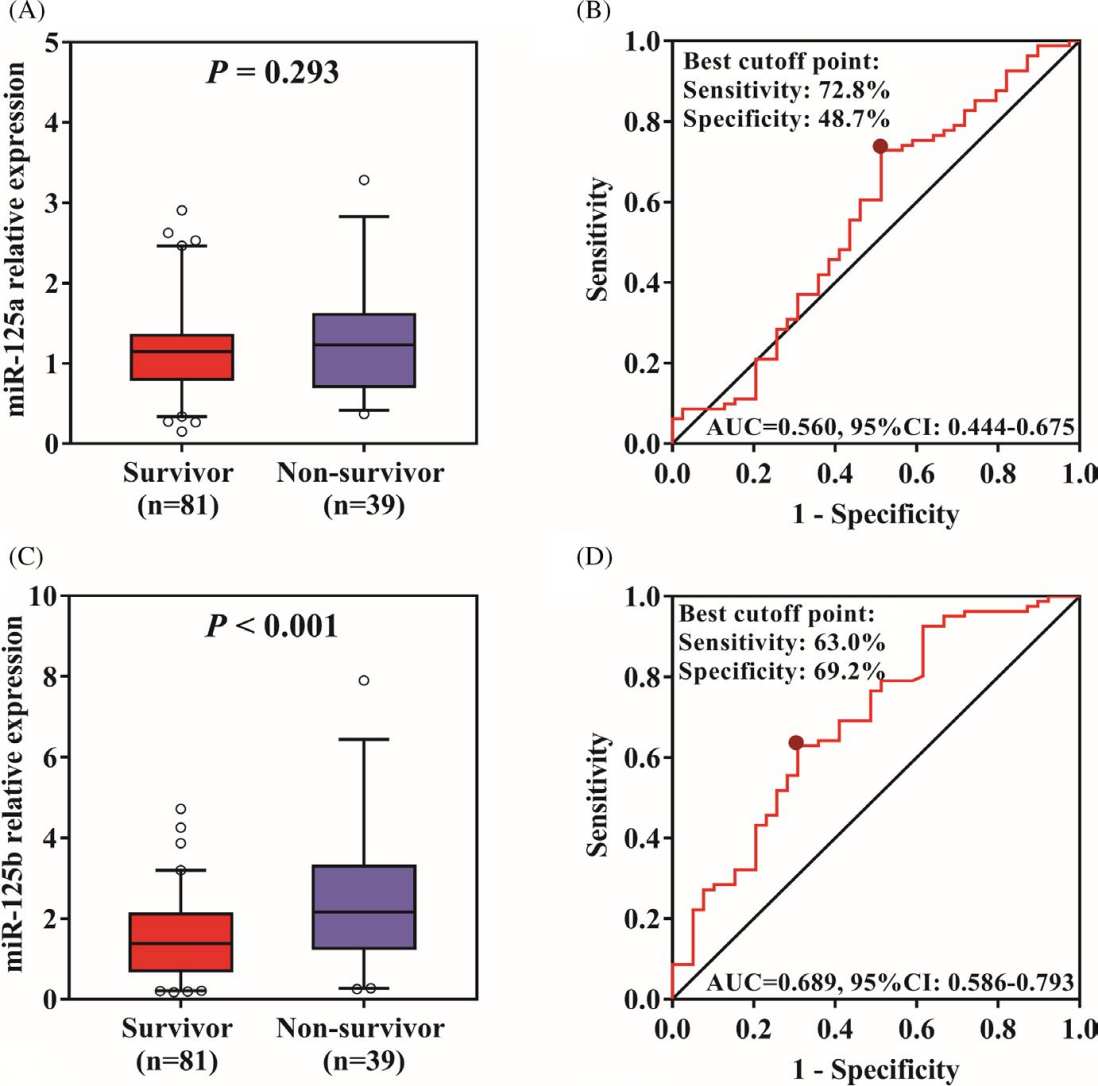
Correlation of miR‐125a/b expression with sepsis mortality. A, miR‐125a expression in survivors and non‐survivors. B, The value miR‐125a expression in predicting mortality. C, miR‐125b expression in survivors and non‐survivors. D, The value miR‐125b expression in predicting mortality

There were 81 survivors and 39 non‐survivors in sepsis patients. MiR‐125a expression showed no difference between survivors and non‐survivors (*P* = .293) (Figure 3A), and ROC curve exhibited miR‐125a could not differentiate survivors from non‐survivors with AUC 0.560 95%CI 0.444‐0.675 (Figure 3B). Importantly, miR‐125b expression was observed to be greatly decreased in survivors compared with non‐survivors (*P* < .001) (Figure 3C), and ROC curve revealed that miR‐125b could well distinguish survivors from non‐survivors with AUC 0.689 95%CI 0.586‐0.793, and the sensitivity and specificity were 63.0% and 69.2% at the best cutoff point (Figure 3D).

### 3.6 Factors predicting mortality in sepsis patients

Univariate and multivariate logistic regression model analysis was further performed to explore factors predicting mortality in sepsis patients (Table 4). Univariate analysis disclosed that miR‐125b expression (*P* = .001), Scr level (*P* = .005), CRP level (*P* < .001), APACHE II score (*P* = .005), SOFA score (*P* < .001), TNF‐α level (*P* = .028), IL‐1β level (*P* = .050), and IL‐17 level (*P* = .018) predicted higher risk of mortality. And further multivariate analysis revealed that miR‐125b expression (*P* = .045), Scr level (*P* = .050), CRP level (*P* = .045), and SOFA score (*P* = .003) were independent factors predicting higher risk of mortality.

**Table 4:** Univariate and multivariate logistic regression model analysis of factors predicting mortality

Items	Univariate logistic regression				Multivariate logistic regression			
	*P* value	OR	95% CI		*P* value	OR	95% CI	
			Lower	Higher			Lower	Higher
miR‐125a relative expression	.193	1.494	0.816	2.733	.882	0.923	0.318	2.679
miR‐125b relative expression	.001	1.849	1.296	2.638	.045	2.074	1.017	4.232
Age	.895	0.998	0.967	1.030	.577	0.984	0.932	1.040
Gender	.898	0.947	0.413	2.174	.471	1.630	0.432	6.143
BMI	.538	0.976	0.904	1.054	.886	1.009	0.890	1.144
Smoke	.581	1.261	0.554	2.870	.778	0.818	0.203	3.299
Chronic comorbidities								
COPD	.252	0.503	0.155	1.631	.194	0.222	0.023	2.154
Cardiomyopathy	.281	0.634	0.277	1.452	.170	0.314	0.060	1.644
Chronic kidney failure	.774	1.208	0.332	4.400	.916	0.902	0.132	6.160
Cirrhosis	.061	0.292	0.080	1.059	.100	0.148	0.015	1.445
Scr	.005	1.545	1.144	2.087	.050	1.655	1.000	2.740
Albumin	.723	0.993	0.953	1.034	.613	0.979	0.904	1.061
WBC	.993	1.000	0.974	1.027	.199	0.968	0.921	1.017
CRP	<.001	1.013	1.007	1.019	.045	1.012	1.000	1.023
PCT	.099	1.019	0.996	1.043	.490	0.983	0.937	1.032
APACHE II score	.005	1.097	1.028	1.170	.087	0.870	0.741	1.021
SOFA score	<.001	1.255	1.109	1.420	.003	1.536	1.158	2.038
TNF‐α	.028	1.003	1.000	1.006	.855	1.000	0.995	1.006
IL‐1β	.050	1.025	1.000	1.051	.595	0.986	0.935	1.040
IL‐6	.182	1.003	0.999	1.007	.148	0.993	0.985	1.002
IL‐17	.018	1.004	1.001	1.007	.203	1.005	0.997	1.014

Note

Factors predicting mortality were determined by univariate and multivariate logistic regression analyses. *P* value <.05 was considered significant.

Abbreviations: APACHE, acute physiology and chronic health evaluation; CI, confidence interval; CRP, C‐reactive protein; IL, interleukin; OR, odds ratio; PCT, procalcitonin; SOFA, sequential organ failure assessment; TNF, tumor necrosis factor.WBC, white blood cell;

## 4 DISCUSSION

In this present study, we observed that: (a) miR‐125b was upregulated and positively correlated with APACHE II score, SOFA score, Scr level, and inflammation level in sepsis patients; (b) miR‐125b was decreased in survivors compared with non‐survivors, and it was an independent factor predicting higher risk of mortality in sepsis patients; (c) miR‐125a lacks potential to be marker for disease risk, progression or prognosis in sepsis patients.

Several published studies have revealed that miR‐125a and miR‐125b might serve as biomarkers for risk for several inflammatory diseases.^11^, ^12^, ^13^ For example, a recent miRNA sequencing study exhibits that plasma miR‐125a and miR‐125b are potential markers for increased risk of acute ischemic stroke.^11^ Another study revealed that colonic mucosal miR‐125b is specially upregulated in ulcerative colitis patients compared with Crohn's disease and HCs, which could serve as a specific marker for ulcerative colitis risk.^12^ In addition, plasma miR‐125b but not miR‐125a was upregulated and correlated with higher risk of acute exacerbation in COPD patients.^13^ These studies suggest that miR‐125b presents with good potential for serving as biomarker for disease risk of several inflammatory diseases, while miR‐125a lacks sufficient evidences. In our study, we observed that miR‐125b but not miR‐125a was upregulated in sepsis patients compared with HCs, and ROC curve revealed that miR‐125b could serve as biomarker for increased risk of sepsis. These might be resulted from that (a) miR‐125b promoted inflammatory cells and mediators via regulating inflammatory signaling pathways such as p38 MARK/NFκB pathway, thus sepsis patients who were featured as severe inflammation presented with increased level of miR‐125b^14^; (b) miR‐125b would induce several organ dysfunctions or failures such as promoting cardiac fibrosis and fibroblast‐to‐myofibroblast transition, and acute‐on‐chronic liver failure, thus sepsis patients who were characterized as multi‐organ dysfunction or failure presented with increased level of miR‐125b.^15^, ^16^

It's also reported that miR‐125a and miR‐125b might be used as biomarker for disease progression or disease severity monitoring, and correlate with systemic inflammation in some diseases.^13^, ^17^, ^18^ For instance, plasma miR‐125a is observed to be negatively correlated with Crohn's disease activity index, CRP level, TNF‐α level, and IL‐17 level in patients with active Crohn's disease.^13^ And plasma exosomal miR‐125b is disclosed to be increased in acute‐phase ischemic stroke patients compared with subacute‐phase ischemic stroke patients.^18^ Furthermore, plasma miR‐125b is exhibited to be positively correlated with TNF‐α level, IL‐8 level, and leukotriene B4 level in patients with acute exacerbation of COPD, and miR‐125b is gradually decreased after treatment, while miR‐125a is not associated with inflammation and does not change after treatment.^13^ These studies imply miR‐125b correlates with advanced disease progression or severity, as well as elevated inflammation in several inflammatory diseases. In this study, we discovered that miR‐125b was positively correlated with APACHE II score, SOFA score, Scr level, and inflammation level in sepsis patients, while miR‐125a showed no correlation with disease severity scores/indexes or inflammation. The possible explanations were that miR‐125b promoted inflammation and organ dysfunction (or failures) via various ways as aforementioned studies,^14^, ^15^, ^16^ thus it positively correlated with disease severity scores and inflammation level in sepsis patients. While due to the double‐edge effect of miR‐125a on regulating inflammation and its related organ injuries, it lacked correlation with disease severity scores/indexes or inflammation. To be noted, we observed that the correlation efficiencies between miR‐125b and key disease severity–related indexes such as inflammatory cytokines, Scr, APACHEII score, etc are weak, thus further study with large samples are needed to validate the results.

As to the prognostic role of miR‐125a and miR‐125b in inflammatory or critical ill diseases, limited data are disclosed. Instead, most previous data focus on the application of miR‐125a and miR‐125b in predicting prognosis in cancers. For instance, reduced miR‐125a level associates with poor survival in hepatocellular carcinoma patients^19^; downregulation of miR‐125b predicts poor response to therapy and short leukemia‐free survival and overall survival in pediatric acute lymphoblastic leukemia patients.^20^ In this present study, we found that miR‐125b but not miR‐125a was decreased in survivors compared with non‐survivors, and it was an independent factor predicting higher risk of mortality in sepsis patients. These might be due to that miR‐125b closely positively correlated with disease severity and inflammation, which resulted in worse outcomes in sepsis patients; while miR‐125a lacked correlation with disease severity scores/indexes or inflammation, thus failed to predict the mortality. Besides, we also explored other independent factors predicting risk of mortality in sepsis patients, and observed that apart from miR‐125b, Scr, CRP level, and SOFA score correlates with increased mortality risk as well. These might result from that increased Scr represented severe renal injury, CRP represented enhanced inflammation, and SOFA reflected comprehensive multiple organ failures, thus they independently predicted increased mortality risk.

Several limitations still existed in this present study. Firstly, the sample size was relatively small; Secondly, the correlation of miR‐125a/b expression with long‐term outcomes was not evaluated; Thirdly, the molecular mechanism of miR‐125a/b in sepsis was not explored.

In conclusion, miR‐125b but not miR‐125a is upregulated and exhibits a trend to correlate with enhanced disease severity, inflammation, and increased mortality in sepsis patients.
